# Supplementary material for: Splice-Junction-Based Mapping of Alternative Isoforms in the Human Proteome
Source: Cell Rep. Author manuscript; Available in PMC 2020 Jan 15. (PMC6961840; doi:10.1016/j.celrep.2019.11.026)
Supplement: 2 [file NIHMS1546469-supplement-2.zip › DF1/PXD010154/AdrenalGland/AdrenalGland_23_HNRNPH1_YVELFLNSTAGASGGAYGSQMLGGMGLCK.pdf]

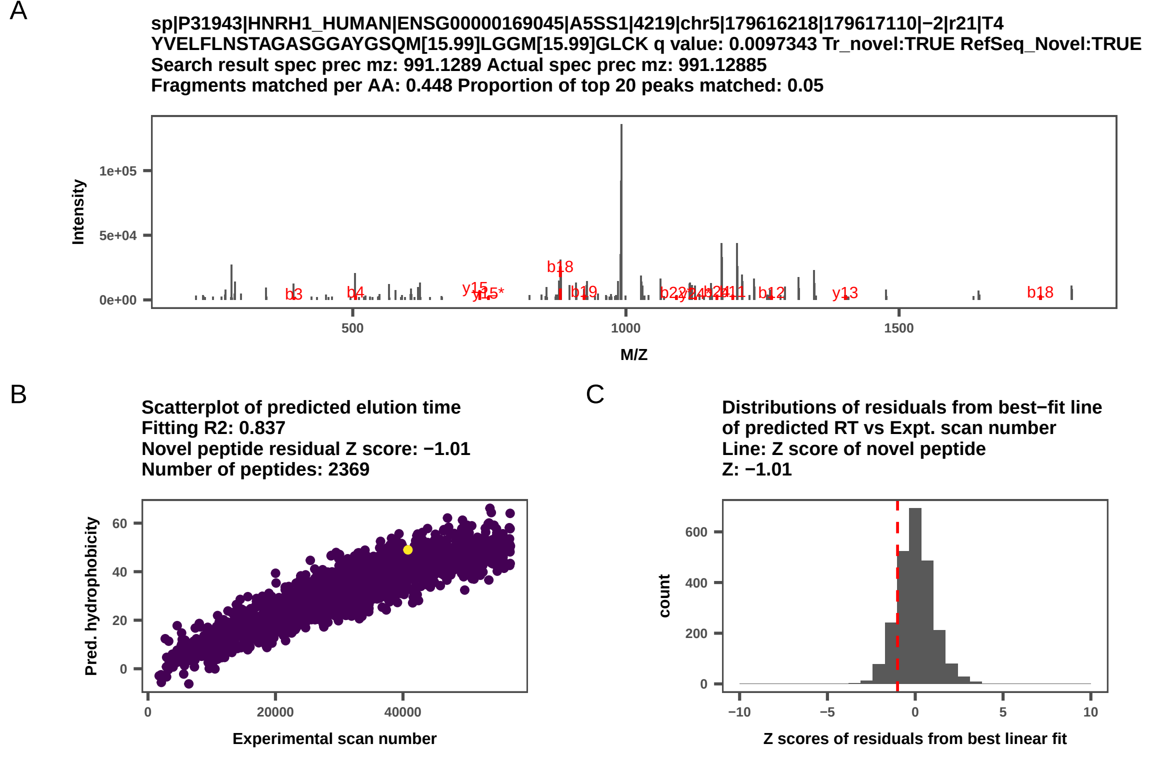
A
sp|P31943|HNRH1_HUMAN|ENSG00000169045|A5SS1|4219|chr5|179616218|179617110|−2|r21|T4
YVELFLNSTAGASGGAYGSQM[15.99]LGGM[15.99]GLCK q value: 0.0097343 Tr_novel:TRUE RefSeq_Novel:TRUE
Search result spec prec mz: 991.1289 Actual spec prec mz: 991.12885
Fragments matched per AA: 0.448 Proportion of top 20 peaks matched: 0.05
Intensity
1e+05
5e+04
0e+00
b3
b4
y15
b18
b19
b12
y13
b18
500
1000
1500
M/Z
B
Scatterplot of predicted elution time
Fitting R2: 0.837
Novel peptide residual Z score: −1.01
Number of peptides: 2369
Pred. hydrophobicity
60
40
20
0
0
20000
40000
Experimental scan number
C
Distributions of residuals from best−fit line
of predicted RT vs Expt. scan number
Line: Z score of novel peptide
Z: −1.01
count
600
400
200
0
−10
−5
0
5
10
Z scores of residuals from best linear fit
